# Application of targeted multi-gene panel testing for the diagnosis of inherited peripheral neuropathy provides a high diagnostic yield with unexpected phenotype-genotype variability

**DOI:** 10.1186/s12881-015-0224-8

**Published:** 2015-09-21

**Authors:** Thalia Antoniadi, Chris Buxton, Gemma Dennis, Natalie Forrester, Debbie Smith, Peter Lunt, Sarah Burton-Jones

**Affiliations:** Bristol Genetics Laboratory, North Bristol NHS Trust, Southmead Hospital, Bristol, BS10 5NB UK; Department of Social & Community Medicine, University of Bristol, Oakfield House, Bristol, BS8 2BN UK

## Abstract

**Background:**

Inherited peripheral neuropathy (IPN) is a clinically and genetically heterogeneous group of disorders with more than 90 genes associated with the different subtypes. Sequential gene screening is gradually being replaced by next generation sequencing (NGS) applications.

**Methods:**

We designed and validated a targeted NGS panel assay including 56 genes associated with known causes of IPN. We report our findings following NGS panel testing of 448 patients with different types of clinically-suspected IPN.

**Results:**

Genetic diagnosis was achieved in 137 patients (31 %) and involved 195 pathogenic variants in 31 genes. 93 patients had pathogenic variants in genes where a resulting phenotype follows dominant inheritance, 32 in genes where this would follow recessive inheritance, and 12 presented with X-linked disease.

Almost half of the diagnosed patients (64) had a pathogenic variant either in genes not previously available for routine diagnostic testing in a UK laboratory (50 patients) or in genes whose primary clinical association was not IPN (14).

Seven patients had a pathogenic variant in a gene not hitherto indicated from their phenotype and three patients had more than one pathogenic variant, explaining their complex phenotype and providing information essential for accurate prediction of recurrence risks.

**Conclusions:**

Our results demonstrate that targeted gene panel testing is an unbiased approach which overcomes the limitations imposed by limited existing knowledge for rare genes, reveals high heterogeneity, and provides high diagnostic yield. It is therefore a highly efficient and cost effective tool for achieving a genetic diagnosis for IPN.

**Electronic supplementary material:**

The online version of this article (doi:10.1186/s12881-015-0224-8) contains supplementary material, which is available to authorized users.

## Background

Inherited peripheral neuropathy (IPN) is the most common group of inherited neurological disorders with an estimated prevalence of 1 in 2500 individuals [1]. It is clinically and genetically heterogeneous; with over 90 genes and loci implicated in the normal function of the myelinated axons of the peripheral nervous system. Onset is typically in the first or second decade, but there are congenital and infantile onset forms of the disease, as well as late onset adult forms. The classical clinical phenotype may manifest as distal limb muscle wasting and weakness, mild to moderate sensory loss, abnormalities of deep tendon reflexes and foot deformities (*pes cavus* and hammer toes). Hearing loss, or respiratory impairment resulting from phrenic nerve involvement, may also be characteristic in some forms.

IPN classification is based on clinical phenotype, mode of inheritance, age of onset, electrophysiological studies and causal mutation. The main subtypes include hereditary motor and sensory neuropathy (HMSN), typically known as Charcot-Marie-Tooth disease (CMT); hereditary sensory and autonomic neuropathy (HSAN) also known as hereditary sensory neuropathy; hereditary motor neuropathy (HMN), also known as distal hereditary motor neuropathy, and hereditary neuropathy with liability to pressure palsy (HNPP).

CMT is the phenotype with the widest genetic heterogeneity. Nerve conduction velocity studies (NCV) subdivide CMT into type 1 (CMT1), a demyelinating form with median or ulnar motor NCV <38 m/s; type 2 (CMT2), an axonal form with NCV >38 m/s, and an intermediate form with both demyelinating and axonal features. Inheritance modes include autosomal dominant (AD), autosomal recessive (AR) and X-linked (XL). A single gene may be implicated in different phenotypes and present with different modes of inheritance, presenting a challenge to diagnose patients with specific types of IPN [2, 3].

Following the exclusion of a 1.5 Mb duplication at 17p11.2 including the *PMP22* gene as the most common cause of CMT1, the traditional strategy for genetic testing consisted of sequential sequencing of individual genes, selected according to the patient’s clinical presentation and family history. This strategy, alongside the cost of serial testing and the limited breadth of genes available for testing, resulted in a low diagnostic yield.

Since the cost of next generation sequencing (NGS) has been decreasing dramatically over the last few years, this technology has found numerous applications in the diagnosis of heterogeneous disorders, including IPN. Whole genome sequencing (WGS) and whole exome sequencing (WES) have identified new genetic causes for many conditions, as demonstrated for IPN by the identification of *SH3TC2* as a cause of autosomal recessive CMT1 [4] and *DYNC1H1* as a new genetic cause for autosomal dominant CMT2 [5].

The targeted panel approach, which restricts analysis to genes known to be implicated in a particular phenotype has also been also successfully applied to IPN. Choi et al. applied WES to a series of unrelated individuals with CMT and restricted analysis to genes already known to be causes of IPN [6]. WGS and WES, however, generate a huge amount of data. Management and storage of this data can present a challenge in a clinical diagnostic environment.

We designed and validated a targeted NGS panel assay including 56 genes associated with known causes of inherited neuropathy and evaluated this approach for the diagnosis of IPN. The results of the pilot project were submitted as a gene dossier to the UK Genetic Testing Network (UKGTN) [7]. This received approval in January 2013 and the diagnostic service was launched in July 2013. We present and summarise the results of 448 patients reported in the first 18 months of this diagnostic service.

## Methods

### Patients

Blood or DNA samples from patients referred by neurologists and clinical geneticists were accepted for testing when the UKGTN approval criteria were met. These criteria are listed below:

- ‘Idiopathic’ peripheral neuropathy diagnosed by clinical presentation with progressive weakness in hands/wrists and/or feet/ankles and/or associated *pes cavus* or finger flexion contractures and/or peripheral sensory loss
- Supportive nerve conduction test result (defining type I or II according to NCV)
- Absence of other non-genetic causes (alcohol, B12 deficiency, diabetes, trauma)
- No associated CNS involvement

The referring clinicians were also asked to indicated the suspected mode of inheritance and provide a recent clinical letter with further details of the clinical phenotype. A small number of samples were accepted for testing from patients not strictly meeting these criteria, after discussion with the individual requesting clinician.

From July 2013 to December 2014, DNA samples from 448 unrelated probands with suspected IPN were tested and reported; a significant proportion of these patients had previously tested negative for the common causes of IPN.

Two hundred ninety nine patients were referred by neurologists (67 %) and 149 patients (33 %) by clinical geneticists. Approximately one third of the patients were under 18 years of age at referral (135 patients, 30 %).

Informed consent for IPN multi-gene panel testing was obtained from patients or their parents/legal guardians by the requesting clinician. The decision to request this test was made by each clinician according to their local ethical guidelines.

This diagnostic test has been assessed for validity, utility and socio-legal/ethical implications in the process of ratification by the UKGTN and UK NHS commissioners, and was undertaken in an accredited UK NHS Laboratory. Data presented pertains only to results of routine diagnostic testing; therefore this study was not subject to ethical approval.

### Targeted capture

Genes were selected following extensive searches of the literature and locus specific databases [8, 9] to ensure their clinical validity and utility. Two additional genes flanking *PMP22* that are commonly involved in the 1.5 Mb reciprocal deletion/duplication event occurring at 17p11.2 [10] were included, to assist in the copy number assessment of this region (*COX10, TEKT3*). All the genes had a disease OMIM (Online Mendelian Inheritance in Man) entry related to a subtype of peripheral neuropathy. Table 1 details the 56 genes included in the assay.

**Table 1:** Genes included in IPN NGS panel, with associated phenotype and inheritance pattern

GENE	OMIM	Locus	CMT1	CMT2	HMN	HS(A)N	OMIM	Inheritance
*AARS*	601065	16q22		CMT 2N			613287	AD
*ARHGEF10*	608136	8p23	Slowed NCV; hypomyelination				608236	AD
*ATL1*	606439	14q11-q21				HSN 1D	613708	AD
*ATP7A*	300011	Xq12-q13			dSMAX3		300489	XL
*BAG3*	603883	10q26.11	Myopathy; myofibrillar, BAG-3 related				612954	AD
*BSCL2*	606158	11q12.3			HMN 5		600794	AD
*CCT5*	610150	5p15.2				HSN with spastic paraplegia	256840	AR
*CTDP1*	604927	18q23	CCFDN: Congenital cataracts, facial dysmorphism, neuropathy				604168	AR
*DCTN1*	601143	2p13.1			HMN 7B		607641	AD
*DNM2*	602378	19p13.2		CMT DI B			606482	AD
				CMT 2 M				
*DYNC1H1*	600112	14q32.31		CMT 2O	SMA-LED		614228/158600	AD
*EGR2*	129010	10q21.1-q22.1	CMT 1D				607678	AD
			CMT 4E CHN				605253	AD
			DSS				145900	AR
*FAM134B*	613114	5p15.1				HSAN 2B	613115	AR
*FGD4*	611104	12p11.21	CMT 4H				609311	AR
*FIG4*	609390	6q21	CMT 4J				611228	AR
*GAN*	605379	16q23.2	Giant Axonal Neuropathy 1				256850	AR
*GARS*	600287	21q22.11		CMT 2D	HMN 5		601472/600794	AD
*GDAP1*	606598	8q21	CMTA RI				608340	AR
			CMT 4A				214400	AR
				CMT 2H			607706	AR
				CMT 2K			607831	AD
*GJB1*	304040	Xq13.1	CMT X1				302800	XL
*HOXD10*	142984	2q31.1	HMSN with Congenital vertical talus				192950	AD
*HSPB1*	602195	7q11		CMT 2F	HMN 2B		606595/608634	AD
*HSPB3*	604624	5q11.2			HMN 2C		613376	AD
*HSPB8*	608014	12q24		CMT 2L	HMN 2A		608673/158590	AD
*IGHMBP2*	600502	11q13.3			HMN 6		604320	AR
*IKBKAP*	603722	9q31.3				HSAN 3	223900	AR
*KARS*	601421	16q23.1	CMT RI B				613641	AR
*KIF1B*	605995	1p36.22		CMT 2A1			118210	AD
*LITAF*	603795	16p13.3-p12	CMT 1C				601098	AD
*LMNA*	150330	1q22		CMT 2B1			605588	AR
*LRSAM1*	610933	9q33.3		CMT 2P			614436	AD/AR
*MED25*	610197	19q33.13		CMT 2B2			605589	AR
*MFN2*	608507	1p35-36		CMT 2A2			609260	AD
				HMSN6			601152	
*MPZ*	159440	1q22	CMT 1B				118220	AD
			CHN				605253	
			CMT DI D				607791	
				CMT 2I			607677	
				CMT 2J			607736	
*MTMR2*	603557	11q21	CMT 4B1				601382	AR
*NDRG1*	605262	8q24.22	CMT 4D				601455	AR
*NEFL*	162280	8p21	CMT 1F	CMT 2E			607734/607684	AD
*NGFB*	162030	1q13.2				HSAN 5, absence of pain	608654	AR
*NTRK1*	191315	1q23.1				HSAN 4; anhidrosis, insensitivity to pain	256800	AR
*PLEKHG5*	611101	1p36.31	CMT RIC		dSMA 4		615376/611067	AR
*PMP22*	601097	17p11.2	CMT 1A				118220	AD
			HNPP				162500	AD
			CMT 1E				118300	AD
			DSS				145900	AR
*PRPS1*	311850	Xq22.3	CMT X5				311070	XL D/R
*PRX*	605725	19q13.1-q13.2	CMT 4F/DSS				145900	AR
*RAB7A*	602298	3q21.3		CMT 2B		HSN	600882	AD
*REEP1*	609139	2p11.2			HMN5B		614751	AD
*SBF2*	607697	11p15.4	CMT 4B2				604563	AR
*SCN9A*	603415	2q24.3	Absence of pain				243000	AR
			Small fibre neuropathy				133020	AD
*SEPT9*	604061	17q25.2-q25.3	Hereditary neuralgic amyotrophy, HNS, HNA & dysmorphic features				162100	AD
*SH3TC2*	608206	5q32	CMT 4C				601596	AR
*SLC12A6*	604878	15q14	PN with agenesis of the corpus callosum				218000	AR
*SOX10*	602229	22q13.1	PCWH syndrome				609136	AD
*SPTLC1*	605712	9q22.1-q22.3				HSAN 1	162400	AD
*SPTLC2*	605713	14q24.3				HSAN 1C	613640	AD
*TDP1*	607198	14q32.11	Spinocerebellar ataxia, with axonal neuropathy				607250	AR
*TRPV4*	605427	12q24.1		CMT 2C			606071	AD
*WNK1*	605232	12p13.33				HSAN 2A	201300	AR
*YARS*	603623	1p13.1	CMT DI C				608323	AD

A custom SureSelect (Agilent Technologies) solution-based oligonucleotide target capture assay was designed using the web-based tool eArray (version 7.7). Regions of interest (ROI) were designed to encompass coding regions of all alternate transcripts for each gene. 5’ and 3’ untranslated regions and non-coding exons were also included. Promoter sites were included for *GJB1* and *PMP22,* and also part of *MPZ* intron 1 [11]. Each ROI included 20 base pairs (bp) upstream and 10 bp downstream of the coding exon to capture canonical splicing donor and acceptor sites.

### Library preparation and sequencing

Genomic DNA was extracted from whole blood using the Puregene protocol (Gentra Systems Incorporated), EZ1 DNA Blood kit (Qiagen) or a standard phenol-chloroform extraction. We also received DNA samples extracted in other laboratories. A Qubit 2.0 Fluorometer (Life Technologies) was used to quantify double stranded DNA concentration in genomic DNA samples. Sequencing libraries were prepared according to the manufacturer’s standard protocol; SureSelectXT Target Enrichment System for Illumina Paired-End Sequencing Library Illumina HiSeq and MiSeq Multiplexed Sequencing Platforms Version 1.5, November 2012. Genomic DNA was sheared to a median size of 200 bp using the Bioruptor NGS sonicator (Diagenode). Fragment size was assessed using the Tapestation 2100 Bioanalyzer (Agilent Technologies). Sequencing was performed on a MiSeq instrument (Illumina) using Version 2 reagents, 2x150 paired-end reads in batches of 16 patients’ samples.

Data analysis was performed using an open source in-house pipeline (alignment: BWA; alignment modification and variant calling: GATKv2; variant annotation: Annovar) with hg19 human genome as a reference, and followed the Association of Clinical Genetics Science (ACGS) Practice Guidelines [12]. Viewing of variants and recording of classification evidence was facilitated using Geneticist Assistant software (Soft Genetics). Copy number enumeration was performed for the 17p11.2 region using the CONTRA tool as a component of the analysis pipeline [13]. This was necessary to ensure that the most frequent cause of CMT1/HNPP (and therefore a positive genetic diagnosis) would not be missed if a patient had not been pre-screened for *PMP22* dosage, for reasons of clinical oversight, or an atypical clinical presentation.

The assay was validated using genomic DNA samples from nine patients previously tested in our laboratory; six of these had single nucleotide variants (SNVs) in six different genes (a total of 26 SNVs),previously identified by Sanger sequencing. A further three had the classical deletion or duplication of *PMP22,* identified previously by MLPA dosage analysis. All 26 SNV occurrences and the *PMP22* copy number variants (CNVs) were confirmed using this assay. Using 95 % confidence intervals for the binomial distribution, the sensitivity of this assay was determined to be between 87 and 100 % [14]. To date, all of the (410) variants detected by NGS and followed up by subsequent Sanger sequencing have been confirmed as true positives. Due to lack of CNV positive controls for genes other than *PMP22*, the analysis pipeline has not been validated as capable of CNV detection automatically. Visual checking of CONTRA data is undertaken when one pathogenic variant is detected in a recessive gene. Small insertions and deletions have been detected and confirmed, ranging from 2 bp to whole gene deletions; however this does not exclude the possibility that there are other CNVs present that were not detected. The report of results states clearly that the test has not excluded copy number variation in the genes examined.

### Variant filtering and classification

Variants were managed using Genetic Assistant (SoftGenetics). Classification followed the Association of Clinical Genetics Science Practice Guidelines [15], and all variants were classified into five pathogenicity groups. Table 2 details the criteria applied to classify variants (Class 1: clearly not pathogenic; Class 2: unlikely to be pathogenic; Class 3: unknown significance; Class 4: likely to be pathogenic; Class 5; clearly pathogenic). Variants were filtered according to their frequency; assessment included comparison of frequency data from the database dbSNP (version 142) [16] and the Exome Variant Server (version 6500) [17]. All variants with frequency above 3 % were considered as clearly not pathogenic (Class 1). The remaining variants were further investigated for their clinical significance. Literature searches, the IPNMDB database [8] and our local laboratory database were interrogated. *In silico* analysis was assisted by AlamutVisual (Interactive Biosoftware), which incorporates multiple amino acid substitution and splice-prediction tools.

**Table 2:** Criteria applied in the classification of variants

Class	Pathogenicity	Criteria
5	Clearly pathogenic	1. Reported in the literature as pathogenic with supporting evidence; multiple independent cases, pedigree segregation studies and/or functional analysis *AND*
		2. Phenotype and inheritance pattern in patient correlates with the gene
4	Likely to be pathogenic	1. Not described in the literature, or weak evidence for pathogenicity in published literature; no segregation or functional analysis available *AND*
		2. Phenotype and inheritance pattern in patient correlates with the gene *AND*
		3. Location of variant in gene and pathogenic mechanism are compatible with previously described pathogenic variants in the gene *AND*
		4. Minor allele frequency (MAF) is <1 % in dbSNP (v142) *and* <1 % in Exome Variant Server (EVS, v6500) *AND*
		5. a) Missense variant; conserved amino acid, Polyphen 2 (HumVar) and SIFT concur in predicting deleterious effect *or* three or more of five splice prediction tools^a^ return >10 % difference in splice site prediction value between wild type and variant *OR*
		b) Frameshift or nonsense variant; where expected mechanism is loss of function *OR*
		c) Synonymous or intronic change; nucleotide highly conserved across multiple species and three or more splice prediction tools return >10 % difference in splice site prediction value between wild type and variant
3	Unknown significance (VoUS)	1. Minor allele frequency <1 % in dbSNP (v142) and EVS (v6500) *AND*
		2. Phenotype and inheritance pattern in patient correlates with the gene *AND*
		3. Not described in the literature, or described in literature with inconclusive or no evidence *AND*
		*a) In silico* predictions score variants as Class 4 but phenotype *does not* correlate with the gene *OR*
		*b)* *In silico* predictions are conflicting (for example; conserved amino acid, very low MAF but Polyphen 2 and SIFT predict benign)
2	Unlikely to be pathogenic	1. a) Minor allele frequency is between 1 % and 3 % in dbSNP (v142) and/or between 1 % and 3 % in EVS (v6500) but phenotype and/or inheritance pattern in patient correlates with the gene *OR*
		b) Minor allele frequency is <1 % in dbSNP and EVS but phenotype and/or inheritance pattern in patient does not correlate with gene *AND*
		2. a) Missense variant; amino acid is weakly conserved across multiple species and/or Polyphen 2 (HumVar) and SIFT concur in predicting benign *OR*
		b) Synonymous or intronic change; nucleotide weakly conserved across multiple species and splice prediction tools^a^ show no significant difference between wild type and variant, even if MAF is <1 % on dbSNP and EVS *AND*
		3. a) Some evidence for benign status in literature but weak or inconclusive *AND/OR* b) Some evidence that variant does not segregate with disease in pedigree of patient(s) tested in this cohort
1	Clearly not pathogenic	Frequency >3 % in dbSNP (v142) or >3 % in Exome Variant Server (v6500) *OR*
		Frequency <3 % but described and proven as not pathogenic in published literature

^a^Five splice prediction tools queried via Alamut software interface: SpliceSite Finder Like, MaxEntScan, NNSplice, GeneSplicer, Human Splicing Finder

Variants classified as pathogenic, likely pathogenic or of uncertain clinical significance were confirmed by Sanger sequencing and were detailed within the report of results. Candidate pathogenic CNVs were confirmed by MLPA analysis either using a commercially available probe mix (MRC Holland), or alternatively by designing bespoke MLPA probes to target the gene of interest, combining these with the MRC Holland P300-A2 reference probe mix. Bespoke MLPA probes were designed using the online tool MAPD [18].

For patients without candidate pathogenic variants, one unique variant was selected and confirmed by Sanger sequencing to ensure the correct identification of all samples in the batch.

## Results and discussion

Analysis of the data demonstrated high read depth and target coverage. On average, 99.81 % of the targeted region was covered to a minimum of 30x reads, and 99.86 % to a minimum of 15x. The mean depth of coverage was 537x reads.

A total of 56,000 variants were detected in the 448 patients. Of those, 1830 variants had prevalence less than 3 % in dbSNP (version 142) or in Exome Variant Server (version 6500). These variants were individually assessed and classified according to the ACGS guidelines [14].

### Gene spectrum in the diagnosis

A total of 195 variants in 31 genes provided a genetic diagnosis for 137 patients (diagnostic yield 31 %). Of these, 107 variants were previously reported in the literature as pathogenic with supporting evidence (Additional file 1: Table S1). The remaining 88 variants were novel and were classified as likely pathogenic (class 4) based on conservation, *in silico* predictions, phenotype compatibility and in several cases family studies (Additional file 2: Table S2). 215 variants were classified as of uncertain clinical significance (Additional file 3: Table S3) and the remaining 1420 variants were assessed as unlikely pathogenic (class 2) or clearly not pathogenic (class 1).

Fifty patients had pathogenic variants in genes not previously available for genetic testing in a diagnostic setting in the UK, including six with variants in regions of the *DYNC1H1* gene not previously screened due to its large size; another 14 had pathogenic variants in genes where testing was previously available but did not feature in the regular IPN diagnostic strategy (Table 3).

**Table 3:** Diagnostic yield for each phenotypic group, mode of inheritance and gene

Phenotype	Inheritance	Gene	Patients
**CMT1**	**AD**	*MPZ*	5
		*NEFL*	3
		*LITAF*	3
		*GDAP1*	3
		*MFN2*	2
		***AARS***	**1**
		*PMP22*	1
	**X-L**	*GJB1*	3
		***ATP7A***	**1**
	**AR**	***SH3TC2***	**10**
		*IGHMBP2* ^a^	4
		***SBF2***	**2**
		***FIG4***	**2**
		***FGD4***	**1**
**Tested**	**101**	**Diagnosed**	**41**
**CMT2**	**AD**	*MFN2*	8
		*BSCL2*	5
		*DYNC1H1* ^b^	4
		*GDAP1*	3
		*HSPB1*	3
		*PMP22*	3
		***SPTLC2***	**2**
		*NEFL*	2
		*SCN9A* ^a^	2
		***GARS***	**2**
		*RAB7A*	2
		*TRPV4*	1
		***SH3TC2***	**1**
		***DNM2***	**1**
		*MPZ*	1
	**X-L**	*GJB1*	6
	**AR**	*IGHMBP2* ^a^	4
		*GDAP1*	2
		***FIG4***	**1**
		***SLC12A6***	**1**
**Tested**	**152**	**Diagnosed**	**54**
**HMN**	**AD**	*DYNC1H1* ^b^	5
		***GARS***	**4**
		*HSPB1*	3
		***REEP1***	**2**
		*MFN2*	2
		*SPTLC1*	1
		***DNM2***	**1**
		*PMP22*	1
		***DCTN1***	**1**
		*NEFL*	1
		*LMNA* ^a^	1
	**AR**	*IGHMBP2* ^a^	2
**tested**	**91**	**Diagnosed**	**24**
**HSN**	**AD**	***SPTLC2***	**1**
		***AARS***	**1**
		*PMP22*	1
		*RAB7A*	1
	**AR**	***MED25***	**1**
**Tested**	**25**	**Diagnosed**	**5**
**Mixed**	**AD**	***AARS***	**5**
		*MPZ*	1
	**X-L**	*GJB1*	2
**Tested**	**22**	**Diagnosed**	**8**
**Complex**	**AD**	*DYNC1H1* ^b^	1
		***SEPT9***	**1**
		***SH3TC2***	**1**
	**AR**	*SCN9A* ^a^	1
		***GAN***	**1**
**Tested**	**57**	**Diagnosed**	**5**
**Overall total**	**448**	**Diagnosed**	**137**

Highlighted bold**:** gene not previously available for routine diagnostic testing in UK

^a^Gene not previously requested for IPN

^b^Only part of the gene was previously available for testing

### Diagnostic yield in the different IPN subtypes

The patients were grouped into a phenotypic subtype according to the information on the clinical proforma provided. Table 3 presents the positive diagnostic yield achieved in detail. The diagnostic yield was highest for patients with demyelinating neuropathy (41/101, 41 %). This cohort included CMT1 patients in whom a 17 p11.2 *PMP22* copy number variant had previously been excluded. The group of the patients with axonal neuropathy had a diagnostic yield of 35.5 % (54/152), similar to the group of patients with mixed neuropathy (36 %, 8/22). The diagnostic yield in patients with HMN was 26 % (24/91) and for HSN 20 % (5/25). Only 5/57 patients received a genetic diagnosis (9 %) in the group with the complex phenotypes. This group included patients with multisystem disorders, where neuropathy was not the primary cause of disease, and UKGTN criteria were not strictly met; however testing was performed as the clinical teams felt it would be beneficial*.*

The majority (93/137, 68 %) of the genetically diagnosed patients had an autosomal dominant form of neuropathy, while 12 patients presented with X-linked disease (9 %). It has been previously estimated that in Northern Europe and North American populations, approximately 90 % of cases of CMT are either autosomal dominant or X-linked [3, 19]. Autosomal recessive disease is estimated to account for significantly less, although in populations with a high rate of consanguineous marriages, autosomal recessive forms can account for up to 40 % [20]. We identified 32 patients with recessive aetiology, representing almost one quarter of our positive cases (23 %). The age of the patients with recessive neuropathy ranged from 3 to 68 years at the time of diagnosis. Fifteen patients were under 18 years of age (47 %) while 17 were adults (53 %). Evidently autosomal recessive peripheral neuropathy is not exclusively associated with very early onset, severe progressive disease. Two genes, *SH3TC2* and *IGHMBP2,* accounted for 62 % of all recessive diagnoses (20/32 patients).

There was no significant difference in the overall diagnostic yield achieved between patients under 18 years of age and those aged 18 and over (36/135 vs 102/313; Fisher’s exact test value 0.222, *p* < 0.05).

The clinical and genetic heterogeneity of IPN has always presented a challenge for the clinical classification. Specialist clinics have played a significant role in guiding the genetic testing. A positive diagnostic yield of 62.6 % in CMT patients attending specialist clinics was reported by Murphy et al. [21] and 67 % by Saporta et al. [22]. This proportion is reported to be significantly lower at 37.7 % in patients that have not been assessed in specialist clinics [21]. These figures include patients positive for the *PMP22* duplication. Our overall diagnostic yield of 31 % does not include *PMP22* duplication positive patients, as the majority of our patients are referred to us for gene panel testing following a normal result for *PMP22* copy number in their local laboratories. For our local patients, the pick-up rate was estimated to be approximately 28 % (125/443) for the *PMP22* duplication and 27 % (90/332) for the *PMP22* deletion (patients tested in a three year period 2007–2010, unpublished data). This figure is slightly higher than the 20.9 % *PMP22* CNVs identified by Murphy et al. [21] in those not attending a specialist inherited neuropathy clinic, and almost twice the level seen in the cohort of DiVincenzo et al., at 14.5 % [23].

DiVincenzo et al. have reported the positive rate of mutations in 14 genes (*PMP22, GJB1, MPZ, MFN2, SH3TC2*, *GDAP1, NEFL, LITAF, GARS, HSPB1, FIG4, EGR2, PRX and RAB7A*) in a very large cohort of 17,789 individuals [23]. Among these patients, 4 genes accounted for 94.9 % of positive results: *PMP22, GJB1, MFN2, MPZ*. Murphy et al. also reported a 94 % pick up rate of those four genes, including point mutations and rearrangements [21]. The equivalent positive pick up rate in our patients, excluding the rearrangements is 43.4 % (we have 36 positive patients in the four genes versus 83 positive in the 14 genes). This possibly reflects the fact that in this first year that the gene panel was available, a significant proportion of the patients referred for testing had already undergone testing for these common genes, and only those without a mutation were referred to us for further testing on the NGS panel.

### Copy number variation

Copy number variation (CNV) is considered rare except for the common 17p11.2 *PMP22* copy number variants, and accounts for about 1 % of diagnoses [24]. We detected two patients with whole *PMP22* gene duplication, and three patients with a whole gene deletion (*PMP22, GJB1, SLC12A6*). We also detected one patient homozygous for *GAN* exon 1 deletion and one compound heterozygous for a partial deletion of the *SBF2* gene encompassing exons 14 to 27.

Our existing pipeline is set up to detect whole gene deletions and duplications; for smaller CNVs we currently manually check the data. However, it has been proven particularly useful to have this ability to check for CNVs in the cases where one pathogenic variant was detected in a gene associated with recessive inheritance.

### The *PMP22* c.353C > T, p.(Thr118Met) variant

We detected the *PMP22* c.353C > T, p.(Thr118Met) variant in five patients (patients 1–5, Table 4). This variant has been widely documented; however its pathogenicity has been controversial in the literature [25–27]. The latest evidence suggested that it is associated with neuropathy, albeit with reduced penetrance [28]. This variant is present in dbSNP (rs104894619) with a minor allele frequency (MAF) of 0.08 %, in the Exome Variant Server with a MAF of 0.53 % (European-American cohort) and in the ExAC browser with MAF 0.73 % (European non-Finnish, including one homozygote). Four out of five of our patients had another class 4 or 5 variant and only one patient had no other variants.

**Table 4:** Interesting patients; examples to highlight the added value of gene panel testing

Patient	Phenotype	Gene and RefSeq Transcript		Variant		Pathogenicity class	Reference
1	CMT2	*PMP22*	NM_000304.2	c.353C > T	p.(Thr118Met)	C5	[25–28]
		*GJB1*	NM_000166.5	c.-17G > A	p.?	C5	[35]
2	CMT2	*PMP22*	NM_000304.2	c.353C > T	p.(Thr118Met)	C5	[25–28]
		*PMP22*	NM_000304.2	c.281delG	p.(Gly94Alafs*17)	C5	[36–38]
3	HMN	*PMP22*	NM_000304.2	c.353C > T	p.(Thr118Met)	C5	[25–28]
		*REEP1*	NM_001164730.1	c.*50G > A	p.?	C5	[39]
		*DYNC1H1*	NM_001376.4	c.3500 T > A	p.(Val1167Glu)	C4	This study
4	HMN	*PMP22*	NM_000304.2	c.353C > T	p.(Thr118Met)	C5	[25–28]
		*GARS*	NM_002047.2	c.485A > G	p.(His162Arg)	C4	This study
5	HSN	*PMP22*	NM_000304.2	c.353C > T	p.(Thr118Met)	C5	[25–28]
6	CMT2	*MFN2*	NM_014874.3	c.1403G > A	p.(Arg468His)	C5	[29–31]
		*MFN2*	NM_014874.3	c.809 T > C	p.(Met270Thr)	C3	This study
		*MFN2*	NM_014874.3	c.1029_1032delGAG	p.(Arg344del)	C3	This study
7	HMN	*MFN2*	NM_014874.3	c.1403G > A	p.(Arg468His)	C5	[29–31]
8	CMT1	*MFN2*	NM_014874.3	c.1403G > A	p.(Arg468His)	C5	[29–31]
9	CMT1	*MFN2*	NM_014874.3	c.2119A > G	p.(Arg707Trp)	C5	[31]
10	CMT2	*PMP22*	NM_000304.2	c.448G > C	p.(Gly150Arg)	C4	This study
11	CMT2	*PMP22*	NM_000304.2	c.(?_-1)_(*1_?) del	p.0	C5	[40]
12	CMT1	*AARS*	NM_001605.2	c.986G > A	p.(Arg329His)	C5	[41]
13	HMN	*PMP22*	NM_000304.2	c.185 T > G	p.(Leu62Arg)	C4	This study
		*MED25*	NM_030973.3	c.1004C > T	p.(Ala335Val)	C5	[42]
14	CMT2	*PMP22*	NM_000304.2	c.(?_-1)_(*1_?) dup	p.(=) dup	C5	[43]
		*SCN9A*	NM_002977.3	c.3369G > T	p.(Leu1123Phe)	C4	This study
15	CMT complex	*PMP22*	NM_000304.2	c.(?_-1)_(*1_?) dup	p.(=) dup	C5	[43]
		*SH3TC2*	NM_024577.3	c.505 T > C	p.(Tyr169His)	C5	[4]
		*MFN2*	NM_014874.3	c.1936G > A	p.(Val646Ile)	C3	This study
16	CMT2	*SH3TC2*	NM_024577.3	c.505 T > C	p.(Tyr169His)	C5	[4]
		*GDAP1*	NM_018972.2	c.501dupA	p.(Glu168Argfs*3)	C4	This study
17	HSN	*RAB7*	NM_004637.5	c.484G > A	p.(Val162Met)	C5	[32]
		*SPTLC2*	NM_004863.3	c.1142 T > C	p.(Phe381Ser)	C4	This study

The diversity of the phenotypes in our patients with the c.353C > T, p.(Thr118Met) *PMP22* variant, the variant’s co-existence with pathogenic mutations in other genes and its high MAF in the general population, challenge it being a causative variant, although its contribution to a phenotype cannot be excluded.

### The *MFN2* c.1403G > A, p.(Arg468His) variant

This variant was detected in three patients (patients 6–8, Table 4). The c.1403G > A, p.(Arg468His) variant is recorded on dbSNP (rs138382758) with MAF 0.20 %, on the Exome Variant Server with MAF 0.24 % (European-American cohort) and in the ExAC with MAF 0.32 % (European, non-Finnish cohort) including two homozygotes. It was originally reported by Engelfried K et al. in a patient with distal weakness and atrophy of the legs and also her symptomatic father, but as it was also detected on one allele in the population study (260 chromosomes), it was considered to be a benign polymorphism [29]. In a later study by Casasnovas et al. the variant c.1403G > A, p.(Arg468His) was identified in six of 14 unrelated Spanish families presenting mild or moderate CMT2, with dominant inheritance and onset of disease in the third to fifth decade [30]. Functional studies were conducted on fibroblasts from a skin biopsy and it was demonstrated that this variant decreased efficiency of ATP synthesis leading to decreased ATP production; this supported the pathogenicity of this variant. The authors suggested that the anonymous control subject identified by Engelfried et al. could be a CMT2 patient who had not yet reached the age of onset of symptoms. Braathen et al. described a patient with CMT1 (reduced NCVs) presenting at age 2, who had the *MFN2* c.1403G > A variant, suggesting that it may also associate with demyelinating CMT [31]. The presence of this variant was consistent with axonal phenotype in two of our patients while the third presented with early onset CMT1, matching the patient described by Braathen et al.

### Broadening the phenotypic spectrum associated with specific genes

A total of six patients were found to have pathogenic variants in genes that would not have been traditionally tested for their phenotype (patients 8–14, Table 4). Two patients referred as having CMT1 were found to have pathogenic variants in *MFN2*. Two patients referred with axonal neuropathy were found to have a *PMP22* variant; one of them the classical *PMP22* deletion and the other the *PMP22* duplication. A patient referred with HMN was also found to have a likely pathogenic variant in *PMP22*, and was carrier of a recessive pathogenic variant in *MED25*. A patient with CMT1 was found to have the recurrent pathogenic variant in *AARS.* Our IPN multigene testing is an unbiased approach; these patients would have not received a diagnosis based on the phenotypically - led genetic testing, either by the traditional sequential testing or by CMT phenotype-specific panels. A few of these patients might have been misclassified in the local clinic, without the benefit of specialist expertise at a regional or national centre; however the NGS panel approach for genetic diagnosis can help to compensate for limited access to specialist diagnostic expertise.

### Identification of multiple genetic causes

Four adult patients were found to have potentially more than one causative variant in different genes (patients 15–17, Table 4). One patient with atypical CMT and known to have the *PMP22* duplication was found to have the known pathogenic variant in *SH3TC2*, c.505 T > C p.(Tyr169His). This patient had also one variant of unknown clinical significance in *MFN2*, c.1936G > A, p.(Val646Ile). Another adult patient with axonal neuropathy was found to have the *SH3TC2* c.505 T > C, p.(Tyr169His) variant alongside a likely pathogenic (predicted truncating) variant in *GDAP1*, c.501dupA, p.(Glu168Argfs*3). While homozygous or compound heterozygous pathogenic variants in *SH3TC2* are associated with autosomal recessive CMT4C, there is growing evidence that heterozygosity for some *SH3TC2* variants can cause axonal neuropathy. Lupski et al. identified the p.(Tyr169His) variant in the heterozygous state in a parent and a grandparent of the proband [4]. The p.(Tyr169His) heterozygotes did not have CMT1, but were found to have patchy axonal polyneuropathy with definite median-nerve mononeuropathy at the wrist on neurophysiology. A patient referred with CMT2 was found to have the *PMP22* duplication and a *SCN9A* likely pathogenic variant c.3369G > T, p.(Leu1123Phe). Another patient presenting with HSN was found to have the known *RAB7A* pathogenic variant c.484G > A, p.(Val162Met) [32], and a likely pathogenic variant in *SPTLC2*, c.1142 T > C, p.(Phe381Ser). These results support the effectiveness of multi-gene testing, since traditional or phenotype-led testing would not have picked up these variants, and have implications for recurrence risk and genetic counselling.

Focused genetic testing has been supported as an approach to provide diagnosis either in the form of small panels or as a tiered approach, by exclusion of variants in common genes before proceeding to NGS testing. The review and recommendations set out by Murphy et al. and Saporta et al. provided essential guidance for clinicians navigating a sea of individual genes in pursuit of a genetic diagnosis [21, 22]. Elsewhere the approach of CMT phenotype-specific panel testing has been proposed for CMT diagnosis as a means to avoid high cost and difficulty in result interpretation [33]. In our experience NGS is efficient and removes the need for serial gene sequencing in most cases. Testing specific genes according to phenotype association may be beneficial if the patients have a very definite neuropathy subtype *and* are referred from expert neurology clinics; for example *GJB1* in a clearly X-linked pedigree, or *PMP22* in HNPP. The cost of design and validation for one large panel is significantly lower than the sum costs of multiple smaller panels. The challenge of variant interpretation should not be underestimated; however prioritisation of variants according to phenotypic compatibility, and the use of *in silico* tools and public databases has proven to be an efficient approach. By incorporating all 56 genes in one assay, we revealed mutations in genes that would have not emerged had we been limited to a phenotype-derived subset of genes. Other authors have also recently been advocating the benefits of expanded multi-gene testing. Hoyer et al. have reported findings similar to ours, including detection of dual pathology and CMT1 patients with *MFN2* mutations [34].

## Conclusions

We developed a 56-gene IPN NGS targeted panel assay as a specialist UK Genetic Testing Network service. This is a frontline diagnostic tool and has largely replaced single-gene Sanger sequencing. Testing was completed for 448 patients in the first 18 months post launch. Genetic diagnosis was achieved in 137 patients (31 %). Testing revealed high heterogeneity, dual pathology and less-tight phenotype-genotype associations.

Assessment and classification of variants is currently a time consuming process, however this task becomes easier as the tools improve and the databases expand. Detailed clinical information is advantageous for variant interpretation, nonetheless we have highlighted cases that would not have achieved a diagnosis had the phenotype been used to guide gene selection.

Clinical-exome or whole-exome sequencing may be appropriate for patients in whom no pathogenic variant is detected on the NGS panel. However, the diagnostic yield achieved at this stage does not support sequencing the exome immediately, despite comparable cost, due to the complexity of analysis of larger data sets. This targeted panel approach has the advantage of producing smaller data sets than exome or genome sequencing, but simultaneously it overcomes the limitation imposed by basing testing decisions on the limited genotype-phenotype data available for the rare genes. It facilitates the testing of category-equivocal cases where the patients do not fit in a particular phenotypic subgroup. It is an efficient approach from the perspective of an accredited diagnostic laboratory, as only one assay needs to be validated. Redesign of the panel is relatively straightforward, allowing inclusion of genes newly identified in IPN pedigrees. Genes with very recently established clinical associations tend not to feature on commercial clinical exome panels which are updated infrequently.

The clinical and genetic heterogeneity of IPN makes both diagnosis and genetic counselling quite challenging [21]. The benefits of obtaining a genetic diagnosis include provision of a definite clinical classification (including clarification of equivocal cases) and guidance on prognosis. Furthermore, accurate genetic risk assessment and cascade testing is beneficial not only for the patient but also for their family. As clinical trials progress for some types of CMT, genotyping will be essential. Advances in sequencing technology have made it time and cost effective to screen large numbers of causative genes simultaneously.

## Additional files

Additional file 1: Table S1. Clearly Pathogenic Variants (Class 5) (XLSX 18 kb) Additional file 2: Table S2. Likely Pathogenic Variants (Class 4) (XLSX 20 kb) Additional file 3: Table S3. Variants of Unknown Clinical Significance (Class 3) (XLSX 33 kb)
